# Insulin topical modulates inflammatory phase and the angiogenesis of the burns wound healing in diabetic-induced rats

**DOI:** 10.1186/1758-5996-7-S1-A259

**Published:** 2015-11-11

**Authors:** Flávia Figueiredo Azevedo, Carla Roberta Oliveira Carvalho, Mário José Abdalla Saad, Maria Helena Melo Lima, Jéssica Da Silva Cunha Breder

**Affiliations:** 1 Universidade Estadual de Campinas, Brazil

## Background

Burns are wounds caused by exposure to an agent from thermal, electric, radioactive or chemistry origin which presents attenuated inflammatory response, becoming even more compromised when associated to diabetes mellitus. Despite the fact from experimental evidences demonstrating tissue healing and reconstruction acceleration due to topical insulin used in diabetic and control rats with incised wounds and burn wounds in diabetic rats, however there is no information regarding molecular and cellular mechanism of topical insulin upon burn wounds.

## Objective

Our aim was to investigate the chemical mediators involved in inflammation and proliferation of topical insulin on wound healing following 2nd degree burns in diabetic rats.

## Materials and methods

Rats were divided into two groups: diabetic rats treated with placebo (DP), and diabetic rats treated with topical insulin (DI). We induced second-degree cutaneous burn wound in streptozotocin-induced diabetic rats with a 1.0-cm diameter circular mold, 120°C, during 20 seconds. Burned rats received either placebo cream or topical insulin (PI 0705370-3), once a day. At 7th and 14th days after the wound induction, anesthetized animals had seen extraction of skin wound sites for Elisa, immunohistochemistry, imunoblotting analysis and Weigert staining.

## Results

Treatment with topical insulin induced early enhancement of macrophages infiltration in the wound surroundings detected by MCP-1 and F4/80 antibodies at day 7 and increased in KGF expression (p<0.05), when compared to DP. At 14th day after burn induction accompanied by enhanced angiogenesis, detected with VEGF and TGF-β1 antibodies; increased expression of alpha smooth muscle actin, present in mature blood vessels; increased cell proliferation, detected with Ki67 antibody on groups treated with topical insulin (DI), when compared to DP.

## Conclusion

There was also increased elastic fibers deposition along the granulation area. Herein we demonstrated that cutaneous treatment with topical insulin accelerated wound healing in the diabetic group by increases the activity of macrophages, promoting angiogenesis and elastic fibers deposition.

